# Bladder flap creation during cesarean delivery. A systematic review and meta-analysis of randomized controlled trials

**DOI:** 10.1186/s12884-026-08744-8

**Published:** 2026-02-24

**Authors:** Nour A El-Goly, Ahmed Mohamed Maged, Sally El-Attar, Nada Kamal

**Affiliations:** 1 Faculty of medicine, Cairo University, Cairo, Egypt; 2 Department of Obstetrics and Gynecology, Kasr Al-Ainy Hospital, Cairo University, Giza, Egypt

**Keywords:** Cesarean delivery, Bladder flap, Bladder dissection, Bladder injury

## Abstract

**Background:**

The aim of the study is to compare the risks and benefits of bladder flap creation and omission during cesarean delivery.

**Methods:**

The databases search from inception to August 2025 included PubMed, Scopus, Embase, Web Of Science, Cochrane library, and clinicaltrials.gov registration using the keywords cesarean delivery AND bladder flap and their MesH terms yielded 13 studies with 3663 participants. All studies were evaluated through risk of bias assessment, and the quality of evidence was assessed through GRADE analysis.

**Results:**

Microscopic hematuria was evaluated in 6 studies with 2738 participants and revealed an Odds Ratio (OR) of 3.23 with 1.10, 9.42 95% Confidence Interval (CI), P value = 0.04, I2 = 94%.

Intraoperative blood loss was evaluated in 3 studies with 609 participants and revealed a mean difference (MD) of 46.75 with -123.50, 217.00 95% Confidence Interval (CI), P value =0.59, I2 =89%. Hemoglobin drop was evaluated in 7 studies with 2953 participants and revealed a MD of 0.15 with -0.05, 0.35 95% Confidence Interval (CI), P value =0.13, I2 =99%. Total operative time was evaluated in 9 studies with 3170 participants and revealed a MD of 7.93 minutes with 3.21, 12.66 95% Confidence Interval (CI), P value =0.001, I2 =97%.

Incision to delivery time was evaluated in 8 studies with 3131 participants and revealed a MD of 0.52 minutes with 0.45, 0.58 95% Confidence Interval (CI), P value <0.001, I2 =99%.

Duration of hospitalization was evaluated in 4 studies with 2574 participants and revealed a MD of 0.33 days with -0.28, 0.95 95% Confidence Interval (CI), P value =0.28, I2 =100%.

Pain VAS score was evaluated in 5 studies with 1150 participants and revealed a MD of 0.88 with 0.20, 1.55 95% Confidence Interval (CI), P value =0.01, I2 =94%.

The risk of bladder injury was evaluated in 5 studies with 1188 participants and revealed an OR of 2.11 with 0.48, 9.20 95% CI, P value =0.32, I2 =0%.

Postoperative complications were evaluated in 4 studies with 1101 participants and revealed an OR of 1.44 with 0.55, 3.75 95% CI, P value =0.46, I2 =0%.

Time to first defecation was evaluated in 3 studies with 356 participants and revealed a MD of 0.46 days with 0.02, 0.91 95% Confidence Interval (CI), P value =0.04, I2 =99%.

**Conclusion:**

Omission of bladder flap creation during CD is associated with significant shortening of the operative time (high evidence) and incision to delivery time (high evidence) and a significant reduction in microscopic hematuria (low evidence), postoperative pain score (low evidence) and time to 1st defecation (moderate evidence) compared to women subjected to bladder flap creation.

**Trial Registration number:**

CRD42022306980

**Supplementary Information:**

The online version contains supplementary material available at 10.1186/s12884-026-08744-8.

## Introduction

Worldwide, the most commonly conducted surgery is Cesarean delivery (CD). Generally, the rate of CD is about 21% with large variation among different areas with less than 5% in Somalia and more than 50% in Egypt and Brazil. Although CD is associated with lower risk of maternal pelvic floor and fetal birth injuries, it carries a twofold increase in maternal and neonatal morbidity compared with vaginal delivery [1].

During the last years the technique of standard CD has undergone several suggestions to optimize the outcome and minimize costs and complications of the procedure. These suggestions include the need for urinary catheter insertion [2], uterine exteriorization versus insitu repair [3], closure of uterine incision in a single or double layers [4], technique of uterine incision closure [5], suturing or omitting peritoneal closure [6], suturing the subcutaneous tissue versus leaving a subcutaneous drain [1], and different techniques of skin closure [7].

Bladder flap creation defined as the dissection of the urinary bladder from the lower uterine segment was a standard step during CD. This aimed to protect the bladder from any inadvertent injury during the coming steps in the procedure [8].

In 1994, Pelosi and Ortega suggested omission of bladder flap creation with other surgical modifications to achieve simplicity, lower costs, and enhance recovery after CD [9].

After that several trials were conducted to compare the advantages and disadvantages of omission of bladder flap creation with conflicting results. The performance of routine formation of bladder flap varies among obstetricians and institutions. There is a lack of consensus in the existing literature about bladder flap creation, especially regarding long-term sequalae such as adhesion formation. A comprehensive and updated systematic review is therefore warranted to synthesize the current available evidence about the risks and benefits of omission of bladder creation during CD and its effects on maternal and surgery outcomes and establish a clear clinical guidance.

## Objective

Comparing bladder flap creation and omission during cesarean delivery.

## Methods

The PRISMA guidelines protocol were followed to prepare the protocol for this review. The protocol was registered at Prospero (CRD42022306980) on 25/2/2022.

### Eligibility criteria, information sources, search strategy

The search included PubMed, Scopus, Embase, Web Of Science, Cochrane library, and clinicaltrials.gov registration till August 2025 and was conducted by two authors independently. The search keywords included cesarean delivery AND bladder flap and their MesH terms. Details about search strategy are shown in Supplementary table S1.

### Study selection

Selection of the included studies was done following PICOS format designed for research question by 2 authors independently. Population were pregnant women who underwent CD whether primary or repeated one. Intervention was the creation of bladder flap before uterine incision. Comparator was omission of bladder flap creation and direct incision of the lower uterine segment. Outcomes included were the presence of microscopic hematuria, intraoperative blood loss, hemoglobin and hematocrit changes after the operation, total operative time, incision to delivery time, duration of hospital stay, postoperative pain score, and development of complications including bladder injury, urinary tract infection, and urine retention. Only randomized controlled studies with no language limitations were included in our review. Cohort, case control, case reports, case series, editorial opinions, letters to editor, review articles, and studies with incomplete data were excluded from our review.

We used a data extraction sheet that was prepared and approved by all authors. The sheet included names of authors, year of publication, study settings, participants characteristics, sample size, intervention details, and outcomes reported.

The studies included in our review were subjected to risk of bias assessment following the Cochrane Handbook recommendations for Systematic Reviews. Items for evaluation were random sequence generation, allocation concealment, blinding of participants and assessors of outcomes, incomplete data, selective data reporting and other biases [10].

The quality of evidence was evaluated using GRADE analysis which includes assessing the studies number, their risk of bias, inconsistencies reported among different studies, indirectness of data, number of participants, limits of confidence intervals and publication bias [11].

### Statistical analysis

Dichotomous variables overall estimate was calculated using Odds Ratio (OR) and 95% confidence interval (CI) while and the mean differences and its corresponding 95% CI was used to calculate the effect estimates for continuous variables. OR greater than 1 indicates that the exposure or intervention is associated with higher odds of the outcome occurring compared with the reference or control group, suggesting a positive association between exposure and outcome. The magnitude of the OR reflects the strength of this association rather than causality.

The random and fixed effect models were used in case of high and insignificant heterogeneity, respectively. The level of significance for P value and I2 test for heterogeneity was set at 0.05 and 40%, respectively. Statistical analysis was done using the Review Manager (RevMan) version 5.4.1 (The Nordic Cochrane Centre, Cochrane Collaboration, 2020, Copenhagen, Denmark) [11].

## Results

### Study selection, study characteristics

**Fig. 1:**
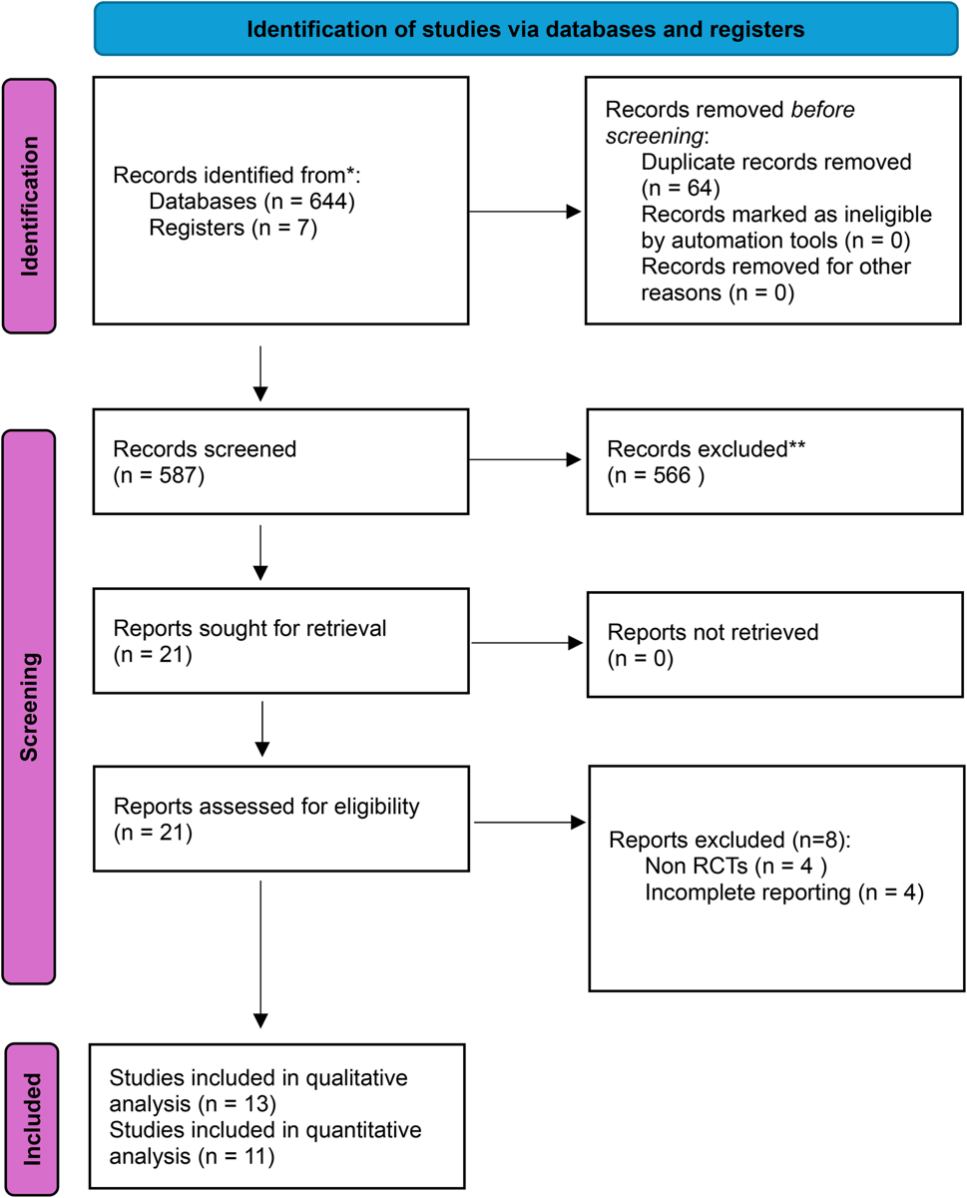
Prisma flow chart

Prisma flow chart of the study is described in Fig. 1.

Thirteen RCTs were included in our qualitative analysis [12–24] but Malvasi and colleagues’ study [19] was not included in quantitative analysis as it was focused on different outcome mainly postoperative adhesion formation and Orji et al. study [22] was excluded from the quantitative analysis as the exact numbers of randomized women could not be reached even after authors contacting.

The included studies were conducted on 3734 randomized participants (only 3663 were analyzed), 1704 of them had bladder flap creation while the other 1749 had bladder flap omission (in 201 women in Orji study, the number for each intervention was not clear). All the studies were conducted in a single center except Akhlaghi 2017 and Malvasi 2011 that were conducted in 2 and 3 centers, respectively. All studies were published in English except Hohlagschwandtner which was published in German. Three studies were conducted in Egypt, 2 in Austria, 2 in Turkey, 2 in USA, and one study was conducted in each of the following countries India, Iran, Italy, and Nigeria. All the studies were not registered except Cetin, O Boyle (retrospective registration), Tuuli, and Youssef. The CD was primary in 8 studies and a mix of primary and repeated CD in 5 studies.

**Fig. 2:**
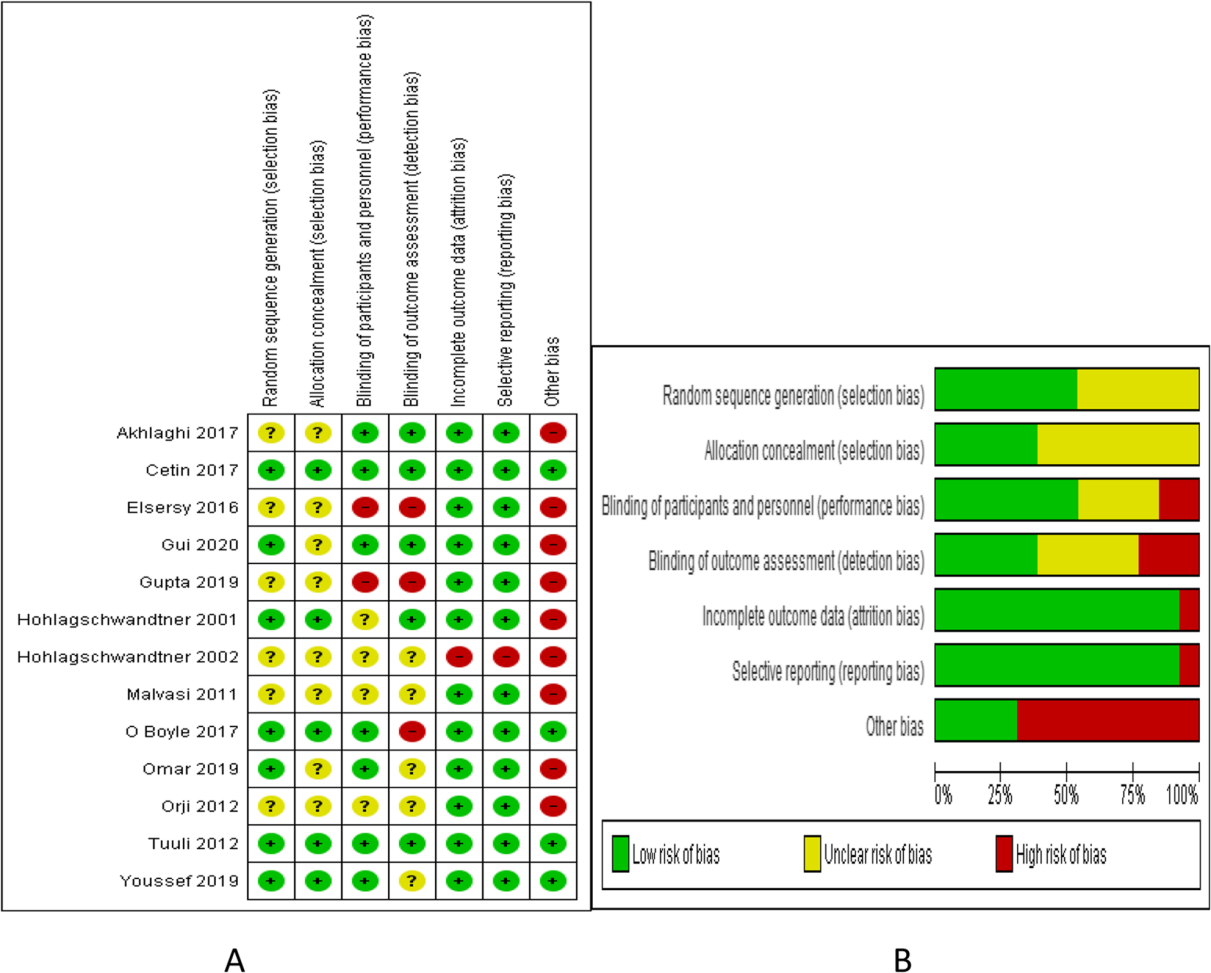
Risk of bias **A** summary **B** graph

The characteristics of the included studies are described in Table 1, and the risk of bias summary and graph is shown in Fig.2.

**Table 1:** Characteristics of the included studies

Study	settings	Design	Size	Participants	Intervention	Outcome
Akhlaghi 2017	Two centers Iran	Not registered	128	Inclusion criteria: CD primary GA ≥ 36 weeks Exclusion criteria: Previous abdominal surgery CD with full cervical dilatation or station zero or lower.	Bladder flap formation (*n* = 64): created through the cutting surface of the visceral peritoneum to isolate the bladder from the lower uterine segment. Avoiding bladder flap formation (*n* = 64): to open the bladder flap, a layer of the peritoneum above the upper edge of the bladder and in the anterior part of the lower uterine segment was taken with forceps in the middle line and transected with the scalpel or scissors. The scissors were placed between the bladder – uterine serous and the lower segment myometrium, and pressed out from the center line, and then while the blades were alternately partially opened, it was withdrawn to isolate a strip up to 2 cm wide from the serous, then the incision was made.	Time to fetal extraction Time of CD Bladder injury Blood loss Hematocrite changes Postoperative pain Hematuria Postoperative complications Duration of hospitalization
Cetin 2017	Single center Turkey	NCT0297787	208 randomized 201 analyzed	Inclusion criteria: Primipara GA > 37 weeks Low risk pregnancy Exclusion criteria: Urinary tract infection Multiple pregnancy EFW > 4000 gm Previous abdominal surgery Associated comorbidities Cervical dilatation > 4 cm	Bladder flap group (*n* = 101):, a bladder flap was created using a scalpel to make a small transverse midline incision in the vesicouterine peritoneal fold, then both index fingers were used to stretch it laterally and caudally to separate the bladder from the low uterine segment. Non-Bladder flap group (*n* = 100) a transverse incision was made 1 cm above the vesicouterine peritoneal fold, and a bladder flap was not created.	Primary outcome Operative time Secondary outcomes: Urinary symptoms Bladder injury Urine retention Postoperative residual urine volume.
Elsersy 2016	Egypt	Not registered	1674	Inclusion criteria: Elective primary and repeat CD GA ≥ 32 weeks. Exclusion criteria: Planned vertical uterine incision Previous abdominal surgeries Sedation and inability to obtain consent	Group 1 (*n* = 838): No Bladder Dissection Group, Uterine incision made 1 cm above the vesicouterine reflection without incision and dissection of the bladder peritoneum. Group 2 Bladder Dissection Group (*n* = 836): Standard CD with incision and dissection of a bladder flap prior to uterine incision.	Primary outcome: Total operating time Secondary outcomes: Times of skin to delivery and to facial closure Blood loss, Hematuria, dysuria, urinary retention Febrile morbidity Hospital stay and readmission Wound infection, Neonatal outcomes
Gul 2020	Single center Turkey	Not registered	100	Inclusion criteria: Age 20–40 years GA 37–42nd weeks Primipara undergoing elective CD Exclusion criteria: Chronic disease (diabetes, hypertension) Previous urinary tract infection Previous laparotomy Emergency cesarean section (abruptio placenta, fetal distress, etc.)	Flap group (*n* = 50): A small transverse midline incision was made with a scalpel at the vesicouterine peritoneum, and the bladder peritoneum was bluntly separated from the uterus using both index fingers. Control group (*n* = 50): A bladder flap was not created; a transverse incision was made 1 cm above the peritoneal vesicouterine layer.	Primary outcome: Changes in urinary symptoms UDI-6 scores Secondary outcomes: Residual urine Hemoglobin changes Urinary symptoms and infection
Gupta 2019	Single center India	Not registered	104	Inclusion criteria: Primipara or multipara with singleton pregnancy with GA≥34weeks Multiparous previous 1 CS with singleton pregnancy with GA≥34weeks. Exclusion criteria: Anomalous baby Previous 2 or 3 CS	Study group (*n* = 54): a low uterine transverse incision was given about 1 to 2 cm above the vesicouterine peritoneal fold without dissection and formation of a bladder flap. Control group (*n* = 50): caesarean delivery was performed in the same way together with the formation of a bladder flap before the uterine incision.	Total operating time Incision-delivery time Pre- and postoperative haemoglobin Urinary tract infection Bowel function Wound healing Hospitalization days Readmissions.
Hohlagschwandtner 2001	Single center Austria	Not registered	102	Inclusion criteria: White women undergoing CD and give their informed consent. Exclusion criteria: fetal malformations Previous uterine surgery	Study group (*n* = 53): A low-transverse uterine incision was performed about 1 cm above the vesicouterine peritoneal fold, without dissection and formation of a bladder flap. Control group (*n* = 49): cesarean delivery was performed in the same way together with formation of a bladder flap before the uterine incision	Total operating time Incision-delivery time Pre- and postoperative hemoglobin Pre- and postoperative urine test for blood Febrile morbidity Postoperative need of analgesics Bowel function Wound healing Hospitalization days Readmissions.
Hohlagschwandtner 2002 German	Single center Austria	Not registered	64	Inclusion criteria: White women undergoing CD and give their informed consent. Exclusion criteria: fetal malformations Previous uterine surgery	Study group (*n* = 31): A low-transverse uterine incision was performed about 1 cm above the vesicouterine peritoneal fold, without dissection and formation of a bladder flap. Control group (*n* = 33): cesarean delivery was performed in the same way together with formation of a bladder flap before the uterine incision	Primary outcome: Hematoma formation Secondary outcomes: Total operating time Incision-delivery time Hemoglobin drop Microhematuria Febrile morbidity Reoperation
Malvasi 2011	3 centers Italy	Not registered	115	Inclusion criteria: Primary CS GA > 38 weeks Exclusion criteria: Previous gynaecological surgery Emergency CD PROM for > 36 h Infections, anticoagulant therapy during pregnancy or delivery Pre-eclampsia, HELLP syndrome, placentae previae, other placental pathologies EFW > 4500 gram	Group 1(*n* = 58): anatomical forceps were used to grasp the visceral peritoneum around the vesico-uterine peritoneal bladder flap. A scalpel was used to make a small transverse midline incision in the VP, and both index fingers were used to stretch it laterally in both directions for approximately 4 cm and caudally for 3 cm in order to separate the bladder from the LUS. Group 2 (*n* = 57): a transverse incision was made 1 cm above the vesico-uterine peritoneal fold. The direct transverse incision of the VP and, subsequently, the myometrium was performed without dissection of the bladder flap.	Primary outcome: Severity of adhesions. Secondary outcomes: Intra-operative blood loss Operative time Bladder injuries, Postoperative urinary dysfunction Postoperative pelvic pain
O’Boyle 2017	Single center USA	Retrospective registration NCT02967913	70 randomized 43 analyzed	Inclusion criteria: Primary elective CD for uncomplicated pregnancy signed an informed consent. Exclusion criteria: Age < 18 years GA < 37 weeks Previous pelvic surgery including cesarean delivery, any surgery involving the bladder, endometriosis, uterine leiomyomata, chronic pelvic pain, or a history of nephrolithiasis during the current pregnancy. CD after failed trial of operative vaginal delivery (forceps or vacuum extraction).	omission of the bladder flap (*n* = 22) and bladder flap (*n* = 21)	The primary outcome was urinary symptom scores using UDI-6 at the 6–8 week postpartum Secondary outcomes: Pelvic floor symptom scores changes Symptom bother responses.
Omar 2019	Single center Egypt	Not registered	550 randomized 514 analyzed	Inclusion criteria: Primary or repeat elective CD GA ≥ 32 weeks Exclusion criteria: Emergent CD Planned vertical uterine incision Previous laparotomies besides CD.	Bladder flap formation (*n* = 236): during CS created through taking a layer of the peritoneum above the upper edge of the bladder and in the anterior part of the lower uterine segment with forceps in the middle line and transected with the scalpel or scissors. Then the scissor placed between the bladder—uterine serous and the lower segment myometrium, and pressed out from the center line, and then while the blades were alternately partially opened, it was withdrawn to isolate a strip up to 2 cm wide from the serous, then the incision was made No bladder flap group (*n* = 278): a low-transverse uterine incision was made approximately 1 cm above the vesico-uterine peritoneal fold without dissection and formation of a bladder flap.	Times of incision, delivery and total CD. Intra- and post-operative complications. Blood loss Hematuria Pain score Need for analgesics Hemoglobin changes Hospital stay Infection
Orji 2012	Single center Nigeria	Not registered	210	Primary CD	No details	intraoperative bladder injury, compare the duration of surgery, compare the occurrence of haematuria in the immediate postoperative period and assess post operative need for analgesics
Tuuli 2012	Single center USA	Prospective registeration NCT00918996	259 randomized 258 analyzed	Inclusion criteria: Primary or repeat CD GA ≥ 32 weeks Exclusion criteria: Emergent CD Planned vertical uterine incision Previous laparotomies besides CD.	Bladder flap formation (*n* = 131): bladder flap was created No bladder flap group (*n* = 127): a low-transverse uterine incision was made approximately 1 cm above the vesico-uterine peritoneal fold without dissection and formation of a bladder flap.	Times of incision, delivery and total CD. Intra- and post-operative complications.
Youssef 2019	Single center Egypt	Prospective registeration NCT03016273	150	Inclusion criteria: Primary or repeat CD GA ≥ 32 weeks Age 18–45 years Exclusion criteria: Planned vertical uterine incision. Previous laparotomies. Patient refusing to participate	Bladder flap (*n* = 75): Dissection of the vesicouterine peritoneal fold to allow bladder retraction. Non bladder flap (*n* = 75): uterine incision made 1 cm above the vesico-uterine reflection without incision and dissection of the bladder peritoneum	Times of operation, incision, delivery, and skin closure. Intraoperative complications. Postoperative Morbidity and mortality Postoperative pain score Need for blood transfusion, Bladder injury Microhematuria.

### Synthesis of results

**Fig. 3:**
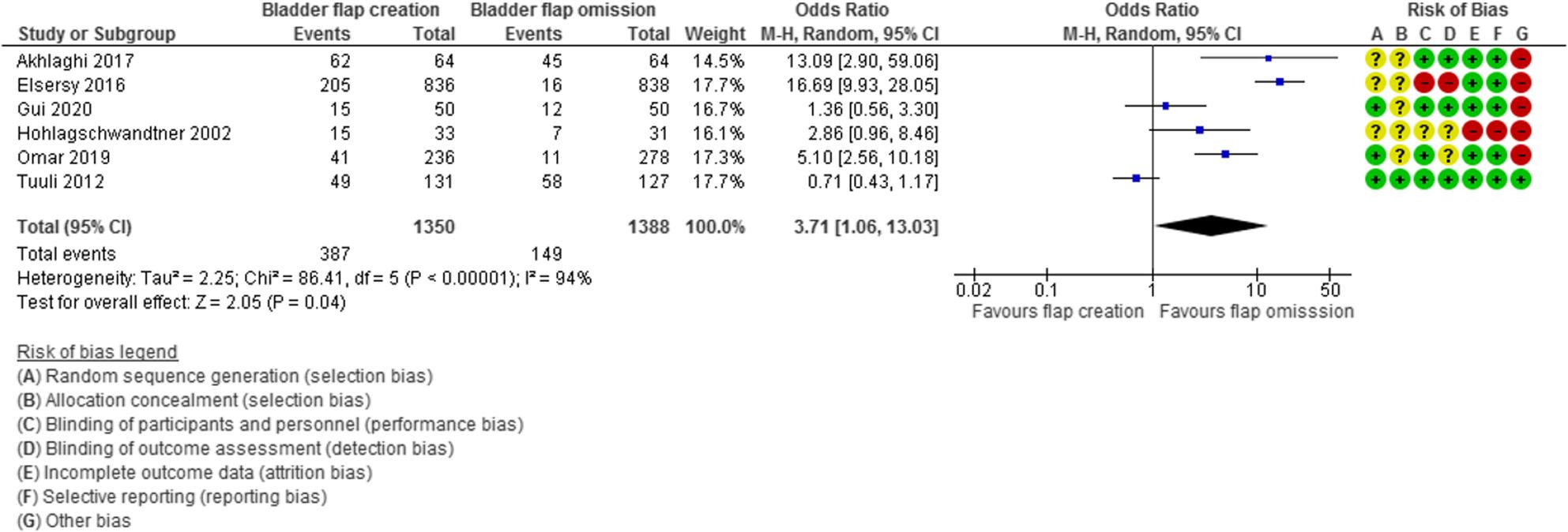
Microscopic hematuria

Microscopic hematuria was evaluated in 6 studies with 2738 participants (1350 subjected to bladder flap creation and 1388 subjected to bladder flap omission) and revealed an Odds Ratio (OR) of 3.23 with 1.10, 9.42 95% Confidence Interval (CI), P value = 0.04, I2 = 94% (Fig. 3).

**Fig. 4:**
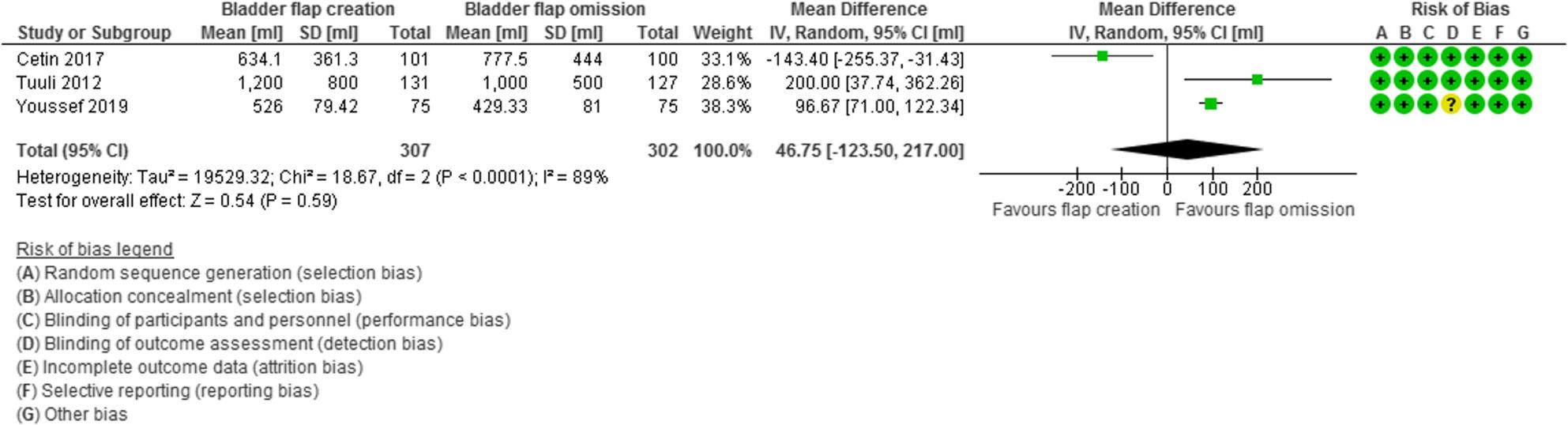
Intraoperative blood loss

Intraoperative blood loss was evaluated in 3 studies with 609 participants (307 subjected to bladder flap creation and 302 subjected to bladder flap omission) and revealed a mean difference (MD) of 46.75 with − 123.50, 217.00 95% Confidence Interval (CI), P value = 0.59, I2 = 89% (Fig. 4).

**Fig. 5:**
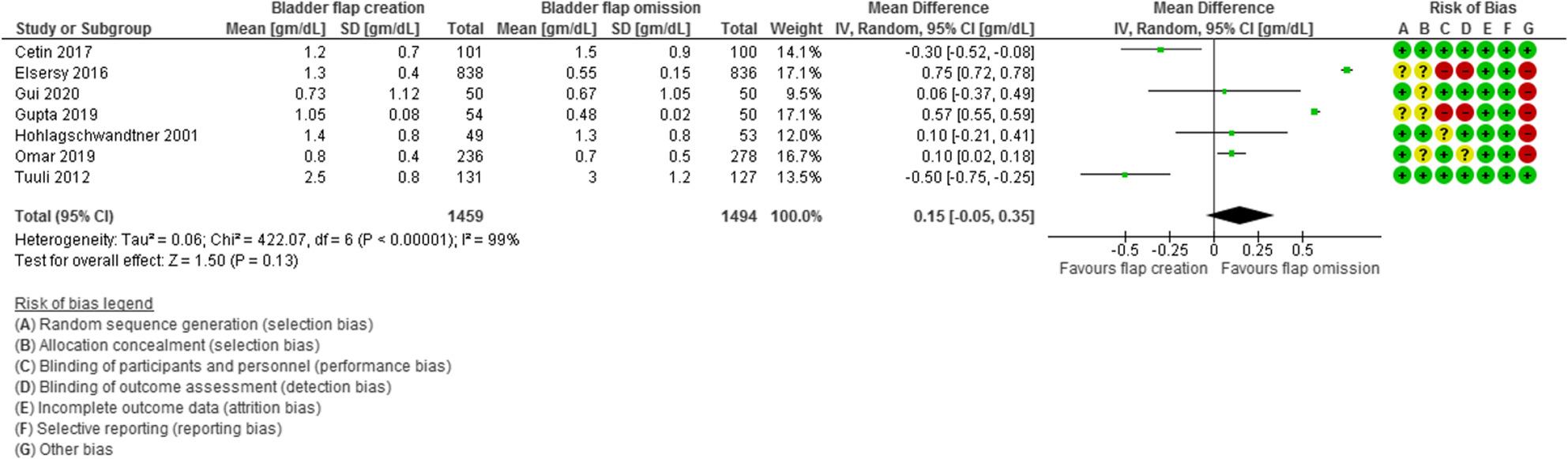
Hemoglobin drop

Hemoglobin drop was evaluated in 7 studies with 2953 participants (1459 subjected to bladder flap creation and 1494 subjected to bladder flap omission) and revealed a MD of 0.15 with − 0.05, 0.35 95% Confidence Interval (CI), P value = 0.13, I2 = 99% (Fig. 5).

**Fig. 6:**
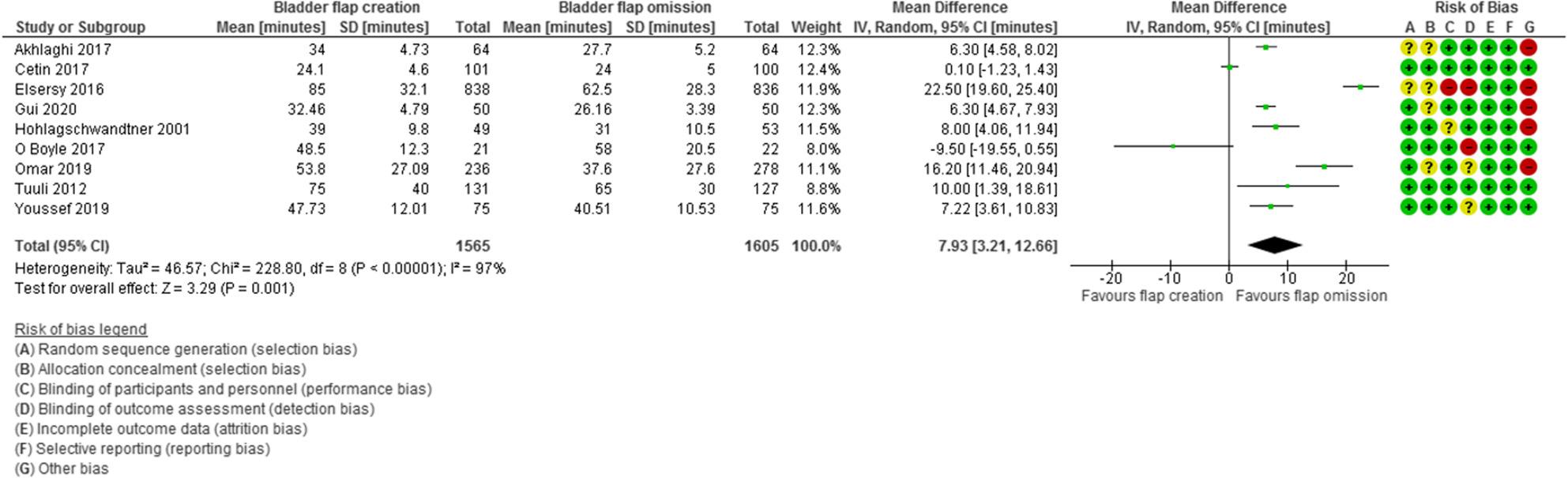
Total operative time

Total operative time was evaluated in 9 studies with 3170 participants (1565 subjected to bladder flap creation and 1605 subjected to bladder flap omission) and revealed a MD of 7.93 min with 3.21, 12.66 95% Confidence Interval (CI), P value = 0.001, I2 = 97% (Fig. 6).

**Fig. 7:**
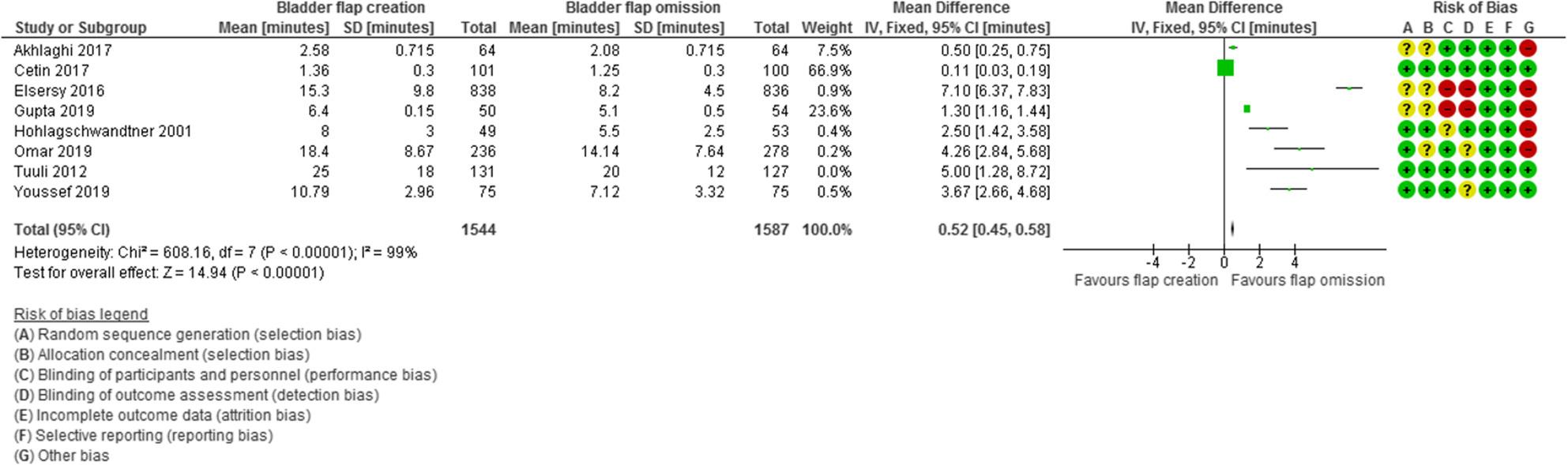
Incision to delivery time

Incision to delivery time was evaluated in 8 studies with 3131 participants (1544 subjected to bladder flap creation and 1587 subjected to bladder flap omission) and revealed a MD of 0.52 min with 0.45, 0.58 95% Confidence Interval (CI), P value < 0.001, I2 = 99% (Fig. 7).

**Fig. 8:**
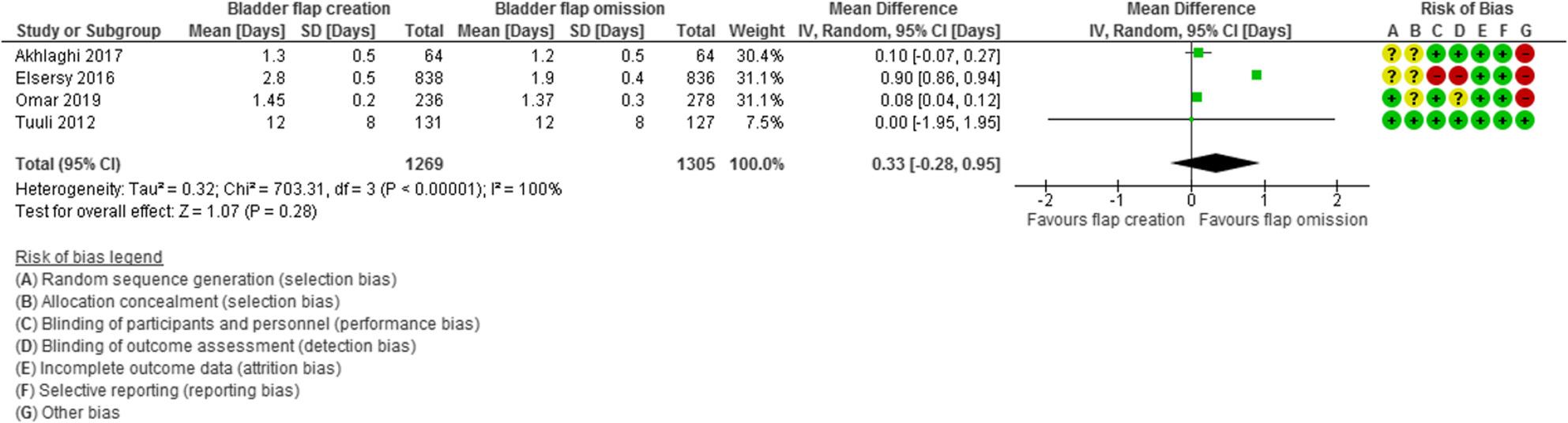
Duration of hospitalization

Duration of hospitalization was evaluated in 4 studies with 2574 participants (1269 subjected to bladder flap creation and 1305 subjected to bladder flap omission) and revealed a MD of 0.33 days with − 0.28, 0.95 95% Confidence Interval (CI), P value = 0.28, I2 = 100% (Fig. 8).

**Fig. 9:**
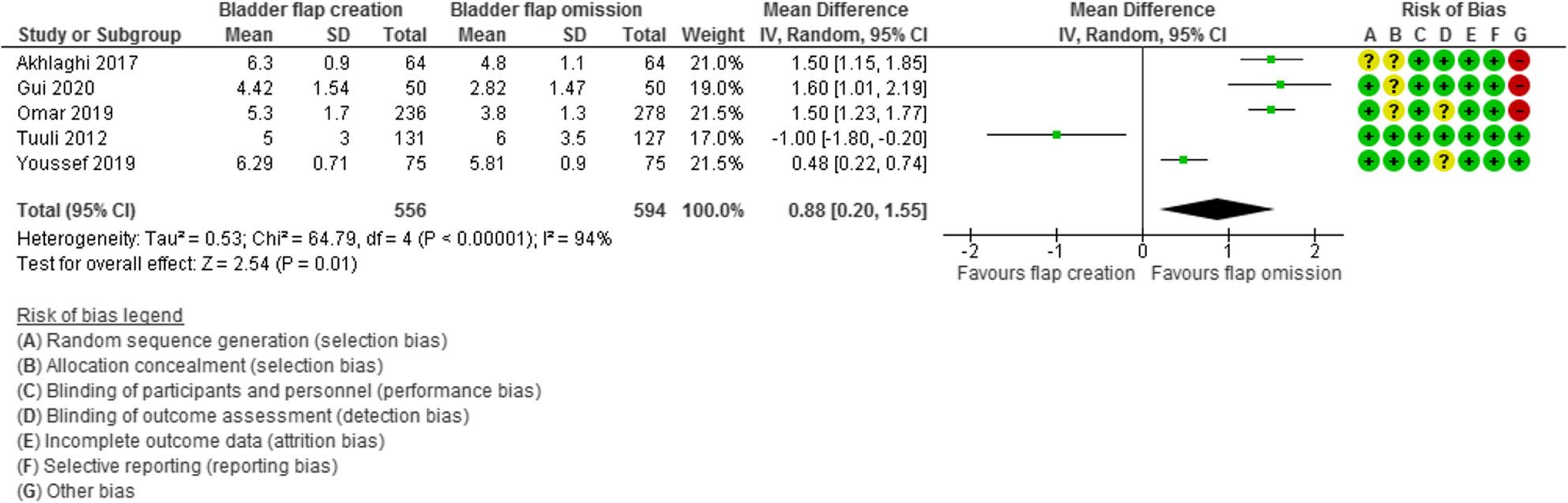
Pain score

Pain VAS score was evaluated in 5 studies with 1150 participants (556 subjected to bladder flap creation and 594 subjected to bladder flap omission) and revealed a MD of 0.88 with 0.20, 1.55 95% Confidence Interval (CI), P value = 0.01, I2 = 94% (Fig. 9).

The risk of bladder injury was evaluated in 5 studies with 1188 participants (576 subjected to bladder flap creation and 612 subjected to bladder flap omission) and revealed an OR of 2.11 with 0.48, 9.20 95% CI, P value = 0.32, I2 = 0%.

Urinary tract infection was evaluated in 3 studies with 876 participants (417 subjected to bladder flap creation and 459 subjected to bladder flap omission) and revealed an OR of 2.20 with 0.53, 9.13 95% CI, P value = 0.28, I2 = 52%.

Time to first defecation was evaluated in 3 studies with 356 participants (174 subjected to bladder flap creation and 182 subjected to bladder flap omission) and revealed a MD of 0.46 days with 0.02, 0.91 95% Confidence Interval (CI), P value = 0.04, I2 = 99%.

Urine retention was evaluated in 2 studies with 316 participants (159 subjected to bladder flap creation and 157 subjected to bladder flap omission) and revealed an OR of 1.25 with 0.18, 8.43 95% CI, P value = 0.82, I2 = 24%.

Postoperative complications were evaluated in 4 studies with 1101 participants (532 subjected to bladder flap creation and 569 subjected to bladder flap omission) and revealed an OR of 1.44 with 0.55, 3.75 95% CI, P value = 0.46, I2 = 0%.

GRADE quality of evidence is shown in Table 2 and subgroup analysis are described in Table 3.

**Table 2:** GRADE quality of evidence

Outcome	No studies	Risk of bias	Inconsistency	Indirectness	Imprecision		Publication bias	Quality
					Sample size	Wide CI		
Microscopic hematuria	6	N	N	N	2738	N	NA	High
Intraoperative blood loss	3	N	S	N	609	S	NA	Low
Hemoglobin changes	7	N	S	N	2953	S	NA	Low
Operative time	9	N	N	N	3170	N	NA	High
Incision to delivery time	8	N	N	N	3131	N	NA	High
Duration of hospitalization	4	N	N	N	2574	N	NA	Moderate
Pain score	5	N	S	N	1150	S	NA	Low
Bladder injury	5	N	N	N	1188	S	NA	Moderate
Urinary tract infection	3	N	N	N	876	S	NA	Low
Time to 1st defecation	3	N	N	N	356	N	NA	Moderate
Urine retention	2	N	N	N	316	S	NA	Low
Postoperative complications	4	N	N	N	1101	S	NA	Low

*N* not serious, *NA* Not applicable, *S* serious

**Table 3:** Subgroup analysis of outcomes

Outcome	Subgroup	Studies	Participants	Effect Estimate	*P* value	I2
Microscopic hematuria	Primary CD	5	612	2.28 [0.80, 6.45]	0.12	83%
	Repeated CD	2	452	2.85 [0.32, 25.68]	0.35	89%
	Primary and repeated CD	1	1674	16.69 [9.93, 28.05]	< 0.001	NA
	Total	6	2738	3.23 [1.10, 9.42]	0.04	94%
Blood loss	Primary CD	2	329	-36.96 [-273.60, 199.69]	0.76	76%
	Repeated CD	1	230	250.00 [47.70, 452.30]	0.02	NA
	Primary and repeated CD	1	150	96.67 [71.00, 122.34]	< 0.001	NA
	Total	3	609	46.75 [-123.50, 217]	0.59	89%
Hemoglobin drop	Primary CD	5	723	-0.04 [-0.18, 0.10]	0.54	45%
	Repeated CD	2	452	-0.36 [-1.19, 0.48]	0.4	94%
	Primary and repeated CD	2	1778	0.66 [0.48, 0.84]	< 0.001	99%
	Total	7	2953	0.15[-0.05,0.35]	0.13	99%
Operative time	Primary CD	7	766	5.67 [2.11, 9.22]	0.002	92%
	Repeated CD	2	580	17.29 [3.79, 30.79]	0.01	85%
	Primary and repeated CD	2	1824	14.90 [-0.07, 29.87]	0.05	98%
	Total	9	3170	7.93 [3.21,12.66]	0.001	97%
Incision to delivery time	Primary CD	5	751	0.18 [0.10, 0.26]	< 0.001	95%
	Repeated CD	2	452	5.81 [4.48, 7.15]	< 0.001	0%
	Primary and repeated CD	3	1928	1.54 [1.41, 1.68]	< 0.001	99%
	Total	8	3131	0.52 [0.45,0.58]	< 0.001	99%
Duration of hospitalization	Primary CD	3	448	0.19 [0.12, 0.25]	< 0.001	0%
	Repeated CD	2	452	0.30 [0.22, 0.38]	< 0.001	0%
	Primary and repeated CD	1	1674	0.90 [0.86, 0.94]	< 0.001	NA
	Total	4	2574	0.33 [-0.28,0.95]	0.28	100%
Pain score	Primary CD	4	548	0.96 [0.27, 1.65]	0.006	85%
	Repeated CD	2	452	-0.04 [-1.74, 1.67]	0.97	89%
	Primary and repeated CD	1	150	0.48 [0.22, 0.74]	< 0.001	NA
	Total	5	1150	0.88 [0.20,1.55]	0.01	94%
Blabber injury	Primary CD	5	736	1.99 [0.29, 13.77]	0.48	21%
	Repeated CD	2	452	3.83 [0.15, 94.65]	0.41	NA
	Primary and repeated CD	0	0	NA	NA	NA
	Total	5	1188	2.11 [0.48,9.2]	0.32	0%
UTI	Primary CD	2	320	1.44 [0.21, 9.93]	0.71	28%
	Repeated CD	2	452	1.55 [0.63, 3.83]	0.34	0%
	Primary and repeated CD	1	104	11.63 [1.42, 95.56]	0.02	NA
	Total	3	876	2.20 [0.53,9.13]	0.28	52%

*CD* Cesarean delivery, *CI* Confidence Interval

Orji and colleagues [22] in their RCT compared the omission of bladder flap against its creation during CD in 210 women. They reported a significant reduction in blood loss (1.9% vs. 11.4%) [*p* = 0.026], duration of operation (43 ± 4.1 min vs. 47 ± 4.7 min) [*p* = 0.001], incision to delivery time (4.6 ± 0.9 min vs. 6.3 ± 1.2 min) [*p* = 0.001], postoperative pain score, needed doses for analgesics [*p* = 0.001], postoperative microscopic hematuria (20% vs. 71.4%) in women with omission of bladder flap compared to those with its creation, respectively. They found no significant differences between both groups regarding post-operative febrile morbidity and wound infection with no reporting of any bladder injury.

Malvasi and colleagues evaluated the value of bladder flap creation on the cesarean scar integrity. Their randomized 115 women undergoing primary CD to either bladder flap creation (58 women) or omission of bladder flap (57 women) and reevaluated them during their second CD. During the repeated CD they took 4 specimens from the scar and examined it by light and electron microscopy. They found higher rates of adhesions in bladder flap formation group compared to omission group (28 (48.3%) (20 mild and 8 severe) vs. 14 (24.1%) (8 mild and 6 severe), respectively). They also found higher rates of submesothelial fibrosis (39.6% vs. 12.2%, *p* < 0.01) and mesothelial stroma neovascularization (46.5%vs 21%, *p* < 0.01), and inflammatory cells (29.7 ± 1.3 vs. 18.3 ± 1.9, *P* < 0.01) in bladder flap creation versus omission, respectively. They concluded that creation of bladder flap is associated with inflammatory, regenerative, and fibrotic processes that can be reduced if bladder flap creation was omitted.

Exclusion of studies at high risk of bias yielded comparable effect estimates, supporting the robustness of the primary analysis (Table 4).

**Table 4:** Sensitivity analysis of outcomes after exclusion of high risk studies

Outcome	Studies	Participants	Effect Estimate	*P* value	I2
Miscroscopic hematuria	4	1000		0.14	90%
Intraoperative blood loss [ml]	3	609	46.75 [-123.50, 217.00]	0.59	89%
Hemoglobin drop [gm/dL]	5	1175	-0.11 [-0.38, 0.15]	0.40	86%
Hematocrite changes	3	271	-0.95 [-2.58, 0.69]	0.26	74%
Operative time [minutes]	8	1496	6.12 [2.68, 9.57]	< 0.001	92%
Incision to delivery time [minutes]	6	1353	0.20 [0.12, 0.28]	< 0.001	95%
Duration of hospitalization [Days]	3	900	0.08 [0.04, 0.12]	< 0.001	0%
Pain score	5	1150	0.88 [0.20, 1.55]	0.01	94%
Complications	4	1101	1.44 [0.55, 3.75]	0.46	0%
Bladder injury	4	1073	5.27 [0.59, 46.93]	0.14	0%
Urine retention	1	201	5.05 [0.24, 106.53]	0.30	NA
Urinary tract infection	2	772	1.55 [0.66, 3.63]	0.32	5%
Time of first defecation	2	252	0.48 [-0.50, 1.46]	0.33	96%

## Discussion

This systematic review and meta-analysis provide a comprehensive and quantitatively robust synthesis of the available evidence comparing bladder flap creation versus omission during cesarean delivery (CD). The findings consistently suggest that routine bladder flap creation confers no meaningful perioperative benefit and may, in fact, be associated with increased operative morbidity, longer operative metrics, and heightened postoperative discomfort.

This meta-analysis found a highly significant shortening of the operative time (high evidence) and incision to delivery time (high evidence) and a significant reduction in microscopic hematuria (low evidence), postoperative pain score (low evidence) and time to 1st defecation (moderate evidence) and a non-significant differences in intraoperative blood loss (low evidence), postoperative hemoglobin changes (low evidence), duration of hospitalization (moderate evidence), risk of bladder injury (moderate evidence), urinary tract infection (low evidence), postoperative urine retention (low evidence) and postoperative complications (low evidence) in women who underwent omission of bladder flap creation compared to those who had creation of bladder flap.

The most clinically salient finding is the significantly increased odds of postoperative microscopic hematuria associated with bladder flap creation. Across six studies encompassing more than 2,700 participants, bladder flap creation was associated with more than a threefold increase in microscopic hematuria compared with omission. Although the confidence interval was wide and heterogeneity was substantial, the directionality of effect was consistent across studies. This observation plausibly reflects increased bladder manipulation and devascularization during flap dissection, leading to mucosal microtrauma or transient ischemia. Importantly, microscopic hematuria, while often self-limited, may represent a surrogate marker of subclinical bladder injury and tissue stress, particularly relevant in high-volume obstetric practice.

In contrast, no significant differences were observed between the two techniques with respect to intraoperative blood loss or hemoglobin drop. These null findings, despite large pooled sample sizes, suggest that bladder flap creation does not meaningfully contribute to hemostatic control during CD. However, the very high statistical heterogeneity (I² > 85% for both outcomes) indicates considerable variability in measurement methods, surgical technique, patient populations, and perioperative protocols. Consequently, these estimates should be interpreted with caution, and the absence of statistical significance should not be conflated with definitive equivalence.

Operative efficiency outcomes strongly favored omission of the bladder flap. Total operative time was significantly prolonged by nearly eight minutes when a bladder flap was created, with a similarly consistent and clinically relevant increase in incision-to-delivery time. Even modest delays in incision-to-delivery interval may carry implications for neonatal outcomes in compromised fetuses and are particularly pertinent in emergency CD settings. The finding of prolonged operative metrics aligns closely with the randomized controlled trial by Orji et al., which demonstrated significant reductions in operative time and incision-to-delivery interval when bladder flap creation was omitted. These data collectively challenge the historical rationale for bladder flap creation as a protective or efficiency-enhancing maneuver.

Postoperative pain outcomes further reinforce the lack of benefit of bladder flap creation. Women undergoing flap creation reported significantly higher pain scores, an effect that is both statistically and clinically relevant. Increased pain likely reflects additional tissue dissection, inflammation, and nerve irritation at the vesicouterine interface. This finding is concordant with Orji et al., who also reported increased analgesic requirements in the bladder flap group. Given current emphasis on enhanced recovery after cesarean delivery, minimizing avoidable sources of postoperative pain is a critical consideration.

Notably, bladder flap creation did not significantly reduce the risk of bladder injury, urinary tract infection, urinary retention, or overall postoperative complications. The absence of a protective effect against bladder injury is particularly important, as avoidance of bladder injury is often cited as the primary justification for bladder flap creation. The pooled estimate showed no statistically significant difference, with low heterogeneity, suggesting that omission of the bladder flap does not compromise surgical safety in this regard. This finding aligns with contemporary surgical principles emphasizing sharp, well-visualized dissection rather than routine prophylactic maneuvers lacking empirical support.

Gastrointestinal recovery, as measured by time to first defecation, was modestly but significantly delayed in the bladder flap creation group. Although the absolute difference was small, it is directionally consistent with increased surgical manipulation and inflammatory response. Duration of hospitalization did not differ significantly between groups; however, this outcome exhibited extreme heterogeneity, likely reflecting institutional policies and non-clinical determinants of discharge rather than true differences in recovery.

Beyond short-term outcomes, the histopathological evidence provided by Malvasi et al. offers compelling mechanistic insight into the potential long-term consequences of bladder flap creation. Their demonstration of increased adhesions, submesothelial fibrosis, neovascularization, and inflammatory cell infiltration in women undergoing bladder flap creation provides biological plausibility for the observed clinical findings. These chronic inflammatory and fibrotic changes may predispose to more difficult repeat cesarean deliveries, increased adhesion-related morbidity, and longer-term pelvic complications. The convergence of histological, clinical, and operative data strengthens the argument against routine bladder flap creation.

There is no available evidence about the use in emergency CD as most of the included studies omitted inclusion of these women, few studies included both elective and emergency CD but the effect on this particular group was not specified. The exclusion of emergency CD in most studies is related to difficulties in randomization properly in the emergency situation.

According to these findings, creation of bladder flap is probably not a necessary step during routine CD. This step can cause unnecessary prolongation of the incision to delivery and total operative time. This prolongation carries a high risk for adverse maternal and neonatal outcomes especially in cases of emergency CD conducted for fetal or maternal distress.

### Strengths and limitations

This is the first comprehensive systematic review to compare the safety and efficacy of bladder flap creation and omission. All the currently available RCTs were included after extensive search efforts. All included studies were subjected to complete data extraction including the risk of bias and all possible outcomes. All outcomes were evaluated for quality of evidence using GRADE analysis. The distribution of studies among different countries including developing and developed countries is another strength of this review.

Several plausible factors may explain this marked heterogeneity. First, substantial clinical heterogeneity exists among the included studies. Variations in patient characteristics—such as parity, number of prior cesarean deliveries, indication for cesarean delivery (elective versus emergency), labor status, degree of bladder descent, and presence of adhesions—are likely to influence operative difficulty, bladder manipulation, and postoperative recovery, thereby affecting outcomes such as operative time, blood loss, pain scores, and urinary symptoms. Importantly, few studies are stratified or adjusted for these key baseline variables.

Second, technical heterogeneity in surgical practice is a major contributor. Bladder flap creation is not a standardized maneuver; its extent, plane of dissection, use of sharp versus blunt techniques, and degree of bladder mobilization varied considerably and were often poorly described. Similarly, comparator intervention may have ranged from minimal bladder handling to partial mobilization in anticipation of uterine incision. Differences in surgeon experience, training background, and institutional cesarean protocols further amplify this variability.

Third, methodological heterogeneity likely played a significant role. Included studies risk of bias, sample size, and outcome ascertainment. Several outcomes were measured using non-uniform definitions and assessment tools; for example, microscopic hematuria was variably defined and inconsistently timed postoperatively, pain was assessed using different VAS scales and at different postoperative intervals, and blood loss was estimated using subjective versus quantitative methods. These inconsistencies inherently inflate heterogeneity and reduce comparability across studies.

Fourth, perioperative care pathways differed widely among studies, including anesthesia type, use of prophylactic antibiotics, bladder catheterization duration, analgesic protocols, and criteria for hospital discharge. Such factors are known to influence pain scores, gastrointestinal recovery, urinary outcomes, and length of stay, yet were rarely controlled for or reported in sufficient detail.

Taken together, the very high heterogeneity observed underscores the need for caution in over-interpreting pooled estimates and highlights the importance of individual high-quality randomized trials with standardized surgical definitions, uniform outcome measures, and transparent reporting. Nonetheless, the consistent direction of effect across multiple clinically relevant outcomes, combined with mechanistic histopathological evidence, suggests that the observed disadvantages associated with bladder flap creation are unlikely to be spurious and warrant serious consideration in contemporary cesarean surgical practice.

Only one study included long term follow up and evaluated adhesion formation mainly. Further subgroup analysis according to technique of CD or type of CD (whether elective or emergency) could not achieved due to limited study numbers. Neonatal outcomes (Apgar score, umbilical cord pH, NICU admission) were not evaluated, despite their known association with incision-to-delivery time. Surgeon experience level was not reported in all studies. Most of the studies were not prospectively registered. Analysis of outcomes may be underpowered to detect rare events.

### Comparison with existing reviews

One metaanalysis assessed the value of bladder flap creation during CD [8]. O’Neill study included 4 RCTs (3 published and 1 unpublished) with 581 participants. They reported that omission of bladder flap creation was associated with shorter incision to delivery interval (WMD 1.27 min; *p* = 0.0001) and associated with no significant differences in the total operative time (WMD 3.5 min), intraoperative blood loss (WMD 42 ml), duration of hospital stay (WMD 0.07 days) or risk of bladder injury (pooled OR 0.96) when compared to women subjected to bladder flap creation. However, this review had limited number of studies with small number of participants. The limited number of studies limited the ability to conduct subgroup analysis. It also lacks assessment of quality of evidence for all outcomes.

## Conclusion

This review suggested that omission of bladder flap creation during CD is associated with significant shortening of the operative time (high evidence) and incision to delivery time (high evidence) and a significant reduction in microscopic hematuria (low evidence), postoperative pain score (low evidence) and time to 1st defecation (moderate evidence) compared to women subjected to bladder flap creation. This omission could be associated with similar intraoperative blood loss, duration of hospitalization, risk of bladder injury, urinary tract infection, postoperative urine retention and postoperative complications to those who had bladder flap creation during their CD. Although the evidence of most outcomes is not high enough to strongly recommend omission of bladder flap creation, we suggest this omission in those women when time is very crucial in the procedure outcomes as those with maternal and or fetal distress. There is lack of high-quality multicenter studies considering the different techniques and indications of CD with adequate long term follow up duration addressing the value of creation or omission of bladder flap. A large RCTs focusing specifically on high-risk groups (e.g., women with prior bladder surgery, dense adhesions, emergency CDs for fetal distress) and studies with long-term follow-up for adhesion-related morbidity conduction is recommended. .

## Supplementary Information

Supplementary Material 1.

## Data Availability

No datasets were generated or analysed during the current study.
