# Supplementary material for: Bladder flap creation during cesarean delivery. A systematic review and meta-analysis of randomized controlled trials
Source: BMC Pregnancy Childbirth. 2026 Feb 24;26:221. doi: 10.1186/s12884-026-08744-8 (PMC12955221; doi:10.1186/s12884-026-08744-8)
Supplement: Supplementary file 1 — Supplementary Material 1. [file 12884_2026_8744_MOESM1_ESM.docx]

Table S1 Search strategy

|  |  |
| --- | --- |
| PubMed | #1 ("Cesarean Section"[Mesh] OR "Cesarean" OR "C-section" OR "cesarean delivery")  #2 ("Bladder"[Mesh] OR bladder OR bladder flap OR bladder reflection OR peritoneal flap)  #3 ("Randomized Controlled Trial" OR randomized OR randomised OR RCT)  #4 #1 AND #2 AND #3 |
| Scopus | TITLE-ABS-KEY ( "cesarean section" OR cesarean OR "C-section" OR "cesarean delivery" )  AND TITLE-ABS-KEY ( bladder OR "bladder flap" OR "bladder reflection" OR "peritoneal flap" )  AND TITLE-ABS-KEY ( randomized OR randomized OR "controlled trial" OR RCT ) |
| Embase | ('cesarean section'/exp OR 'cesarean' OR 'c-section' OR 'cesarean delivery')  AND ('bladder'/exp OR bladder OR 'bladder flap' OR 'bladder reflection' OR 'peritoneal flap')  AND ('randomized controlled trial'/exp OR random* OR RCT) |
| Web Of Science | TS = ( "cesarean section" OR cesarean OR "C-section" OR "cesarean delivery" )  AND TS = ( bladder OR "bladder flap" OR "bladder reflection" OR "peritoneal flap" )  AND TS = ( randomized OR randomised OR "controlled trial" OR RCT ) |
| Cochrane library | #1 MeSH descriptor: [Cesarean Section] explode all trees  #2 (cesarean OR "C-section" OR "cesarean delivery")  #3 (bladder OR "bladder flap" OR "bladder reflection" OR "peritoneal flap")  #4 #1 OR #2  #5 #4 AND #3  #6 filter: Trials |
| Clinicaltrials.gov | Condition/disease Cesarean delivery  Other terms Cesarean section  Intervention/ treatment: (bladder OR "bladder flap" OR "bladder reflection" OR "peritoneal flap")  Study status All studies |
